# Side-branch expansion capacity of contemporary DES platforms

**DOI:** 10.1186/s40001-021-00595-7

**Published:** 2021-10-12

**Authors:** Alper Öner, Paula Rosam, Finja Borowski, Niels Grabow, Stefan Siewert, Wolfram Schmidt, Klaus-Peter Schmitz, Michael Stiehm

**Affiliations:** 1 Department for Cardiology, Center for Internal Medicine, University Medical Center Rostock, Ernst-Heydemann-Straße 6, 18057 Rostock, Germany; 2 Institute for Implant Technology and Biomaterials-IIB E.V, Associated Institute of the University of Rostock, Warnemuende, Rostock, Germany; 3 Institute for Biomedical Engineering, University Medical Center Rostock, Warnemuende, Rostock, Germany

**Keywords:** Bifurcation, Bench test, Mechanical load, Recoil

## Abstract

**Background:**

Percutaneous coronary interventions (PCI) of bifurcation stenoses are both complex and challenging. Stenting strategies share that the stents’ side cells must be carefully explored and appropriately prepared using balloons or stents. So far, stent manufacturers have not provided any information regarding side-branch expansion capacity of their stent platforms.

**Aims:**

Given that drug-eluting stent (DES) information regarding their mechanical capacity of side-branch expansion is not available, we aimed to evaluate contemporary DES (Orsiro, BIOTRONIK AG; Xience Sierra, Abbott Vascular; Resolute Integrity, Medtronic; Promus Premier Select, Boston Scientific; Supraflex Cruz, Sahajan and Medical Technologies) by their side-branch expansion behavior using in vitro bench testing.

**Methods:**

In this in vitro study, we analyzed five commercially available DES (diameter 3.0 mm), measuring their side-branch expansion following inflation of different high-pressure non-compliant (NC) balloons (balloon diameter: 2.00–4.00 mm), thereby revealing the morphological characteristics of their side-branch expansion capacities.

**Results:**

We demonstrated that all tested contemporary DES platforms could withstand large single-cell deformations, up to 4.0 mm. As seen in our side-branch experiments, DES designs consisting of only two connectors between strut rings did not only result in huge cell areas, but also in larger cell diameters following side-branch expansion compared with DES designs using three or more connectors. Furthermore, the stent cell diameter attained was below the balloon diameter at normal pressure.

**Conclusions:**

We recommend that the expansion capacity of side-branches should be considered in stent selection for bifurcation interventions.

## Introduction

Percutaneous coronary interventions (PCI) of bifurcation stenoses are still complex and challenging procedures, because a bifurcation intervention is required in approximately 20% of PCIs [1]. While current guidelines and expert consensus all recommend provisional stenting for managing bifurcation lesions, a two-stent strategy must be considered if the side-branch displays a diameter ≥ 2.5 mm, and if the lesion exhibits significant stenosis within the side-branch ostium [2]. The Medina classification is among the most widespread methods to characterize bifurcation lesions in this regard [3]. Several two-stent strategies can be applied to technically perform a bifurcation stenting, including the crush stenting procedure, culotte stenting, T-stenting, and TAP stenting, along with different variations previously described in the literature [2, 4]. However, all these stenting strategies share that the stents’ side cells must be carefully explored and appropriately prepared using either balloons or stents. So far, stent manufacturers have only specified their stents’ radial expansion capacity. Therefore, we conducted a pilot study designed to analyze stents’ side-branch expansion [5], which has thereby been validated and further expanded. To our knowledge, the side-branch expansion capacity of the different stent models has not been assessed in other studies reported to date.

In the current study, we analyzed several stents, measuring their side-branch expansion following inflation of different high-pressure balloons, thereby revealing the morphological characteristics of their side-branch expansion capacities.

## Material and methods

### Stent models

Five commercially available contemporary stent platforms were investigated: Orsiro 3.00 mm × 15 mm (BIOTRONIK AG, Switzerland), Xience Sierra 3.00 mm × 15 mm (Abbott Vascular, USA), Resolute Integrity 3.00 mm × 15 mm (Medtronic, USA), Promus Premier Select 3.00 mm × 16 mm (Boston Scientific, USA), and Supraflex Cruz 3.00 mm × 16 mm (Sahajanand Medical Technologies, SMT, India). The selected stents represent a mid-size workhorse dimension for coronary vessels. All but the Resolute Integrity stent platform display a so-called slotted tube design, where a strut pattern is laser-cut from metallic tubing. Concerning Resolute Integrity, this stent platform consists of a sinusoidal-waved single strand of cobalt alloy that is laser-fused at different locations, thereby forming an off-phase and open-cell strut pattern. The Orsiro and Resolute Integrity stents exhibit a characteristic helix-shaped strut arrangement, while the other stent platforms are built up from zigzag-shaped, yet circumferential, strut rings.

**Fig. 1:**
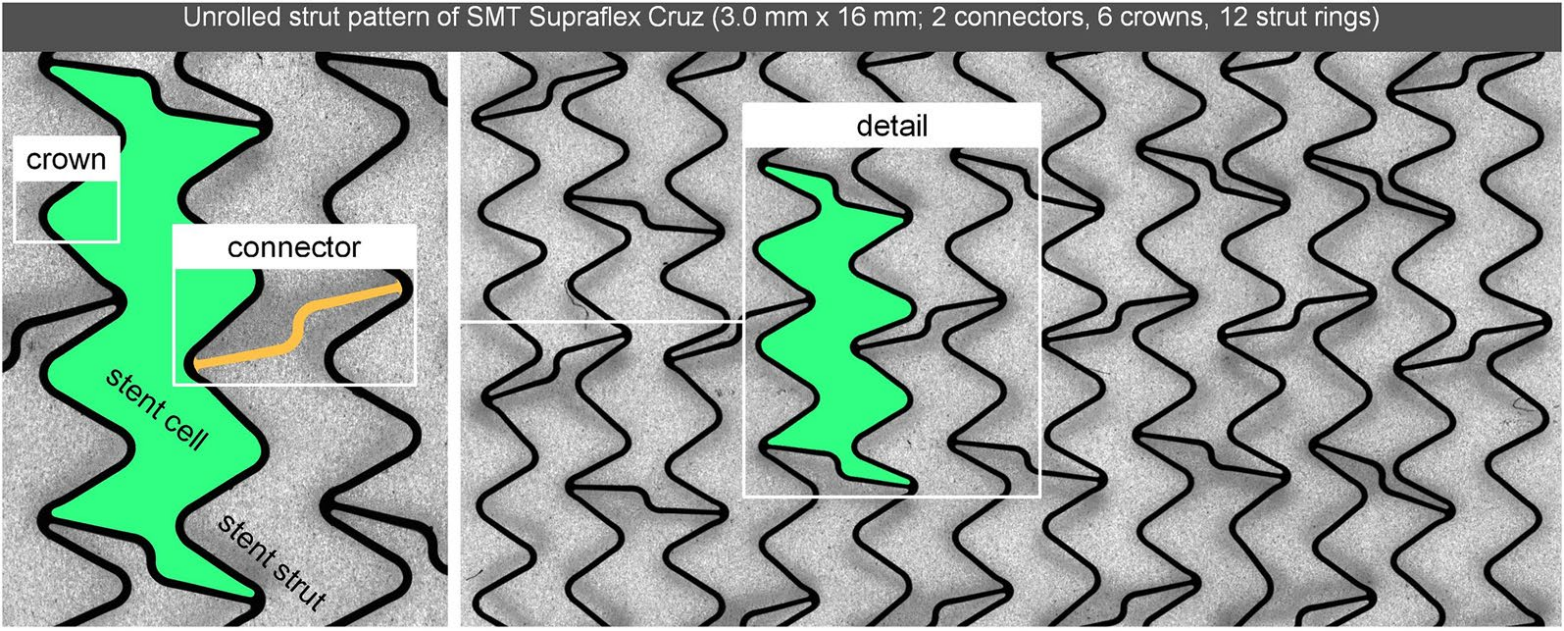
Unrolled strut pattern of SMT Supraflex Cruz (3.0 mm × 16 mm) with labeled stent design features, including stent strut, stent cell, connector, and crown

All stent platforms share the property that their strut rings are linked by means of connectors, which are relevant for the structure’s longitudinal stability [6]. The strut zigzag is defined as crowns or peaks (one peak/crown = two struts), playing a crucial role in stent expansion capacity and radial support, as well [7]. The area surrounded by a pair of connectors and strut rings is defined as a stent cell [7]. Therefore, the number and area of cells are determined by the number of connectors, strut rings, and crowns. Figure 1 illustrates the used stent design nomenclature.

**Fig. 2:**
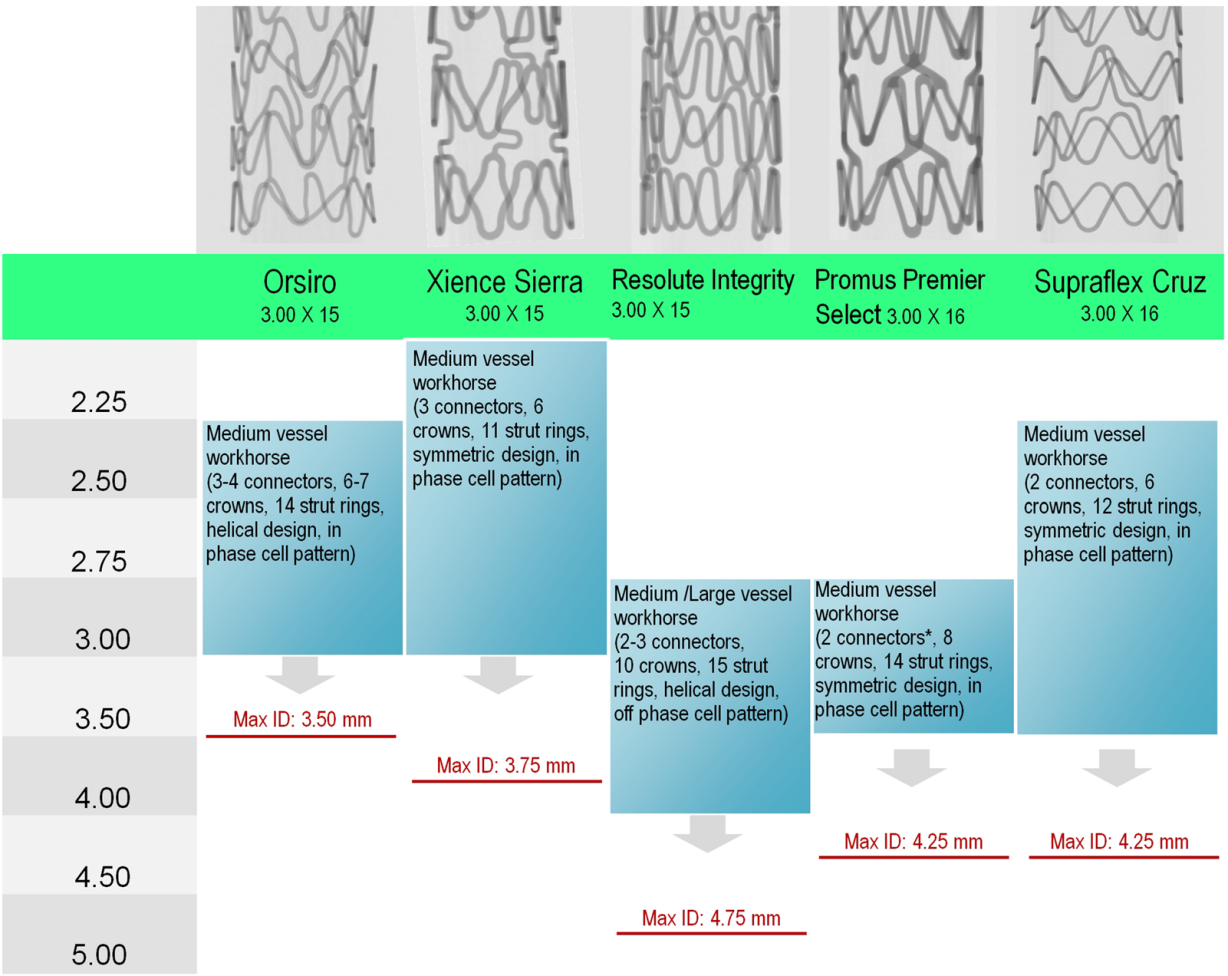
Nominal vessel diameter coverage of the analyzed stent workhorse designs (stent diameter at nominal pressure approx. 3.0 mm), including key design features like the number of connectors, crowns, and strut rings, as well as cell pattern, and post-dilatation limit of inner stent diameter (Max ID), according to the instructions for use (illustration according to [7, 8])

Coronary stents must cover a wide range of target vessel diameters (< 2.0 mm to > 4.0 mm). Therefore, each stent platform is available in a variety of sizes, ranging usually from two to four, which can be adapted to a limited diameter range via different delivery balloons. It is commonly accepted that workhorse stent designs, as those used in this in vitro study, cover the range of medium vessel diameters of approximately 3.0 mm [7] (Fig. 2). To estimate the expansion capacity, the cardiologists must precisely know the cut-off diameter, in addition to the post-dilatation limit of inner stent diameter (Max ID) for a given stent size.

### Stent design measurement

Stent pattern measurements were conducted on one sample of each stent platform. The Finescan Sierra (MKS Instruments) metrology system was employed for acquiring images of the stent platforms in flat projection. As a result, unrolled high-resolution strut patterns were generated without projection errors. Cell area and cell perimeter were measured using the ProAnalyst image analysis software. A minimum of 10 cells were analyzed for each stent, with only full-size cells actually measured. The latter appears crucial for helical stent design (Orsiro and Resolute Integrity), but also for Promus Premier stent consisting of a proximal pattern compression.

### In vitro experiment

**Fig. 3:**
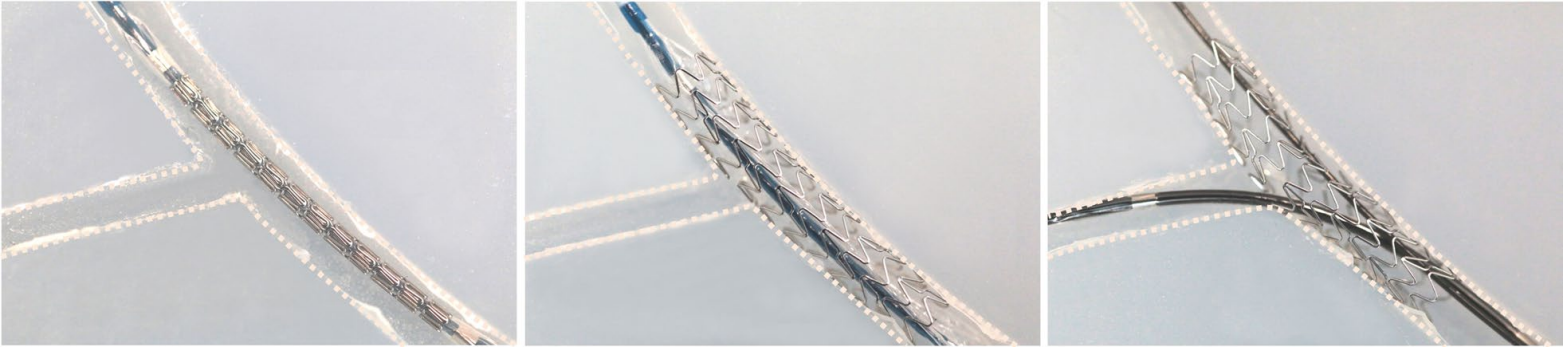
Side-branch expansion in the in vitro experiment using a phantom bifurcation model

Three samples of each of the five commercially available state-of-the-art stent platforms were initially deployed in a phantom bifurcation model with balloons inflated at the nominal pressure (NP), as stated in the manufacturers’ instructions for use (Fig. 3). The stent cell to be tested was determined according to the stent position. The side-branch accessibility of each sample was investigated by means of post-dilatations of one cell using non-compliant balloons (BIOTRONIK Pantera LEO NC) inflated at nominal pressure (NP) = 14 ATM. The balloon size was gradually increased (Table 1). Initially and following each expansion step, cell opening was measured microscopically in the lateral view. Therefore, side-branch experiments were performed without using a phantom bifurcation model. Cell opening was quantified using a circle whose circumference was fitted within the stent cell struts. Several researchers previously described this approach for assessing side-branch accessibility or cell opening in order to assess vessel scaffolding or in the context of bifurcation stenting [9, 10].

**Table 1:** Non-compliant balloons used for post-dilatations to assess side-branch accessibility

Balloon catheter	Dimension [mm]	Balloon pressure NP [atm]	Expected diameter at NP [mm]
Biotronik Pantera LEO	2.00/20	14	2.00
Biotronik Pantera LEO	2.25/20	14	2.25
Biotronik Pantera LEO	2.50/20	14	2.50
Biotronik Pantera LEO	2.75/25	14	2.75
Biotronik Pantera LEO	3.00/30	14	3.00
Biotronik Pantera LEO	4.00/30	14	4.00

On average, three measurements were taken for quantifying cell opening. Stent imaging was performed using Olympus SZX16 microscope. Measurements of the circle within the stent cell were conducted using the Stream (Olympus) calibrated image analysis software, as described previously [9].

Our post-dilatation studies covered the clinically relevant scenarios of a side-branch expansion comprised between 2.0 mm and 3.0 mm. An expansion of 4.0 mm was applied as a benchmark of stent cell overexpansion for a 3.0-mm stent model, without risking strut fracture. Further stent cell expansion by using larger balloons was thereby rendered possible.

## Results

### Stent design

**Fig. 4:**
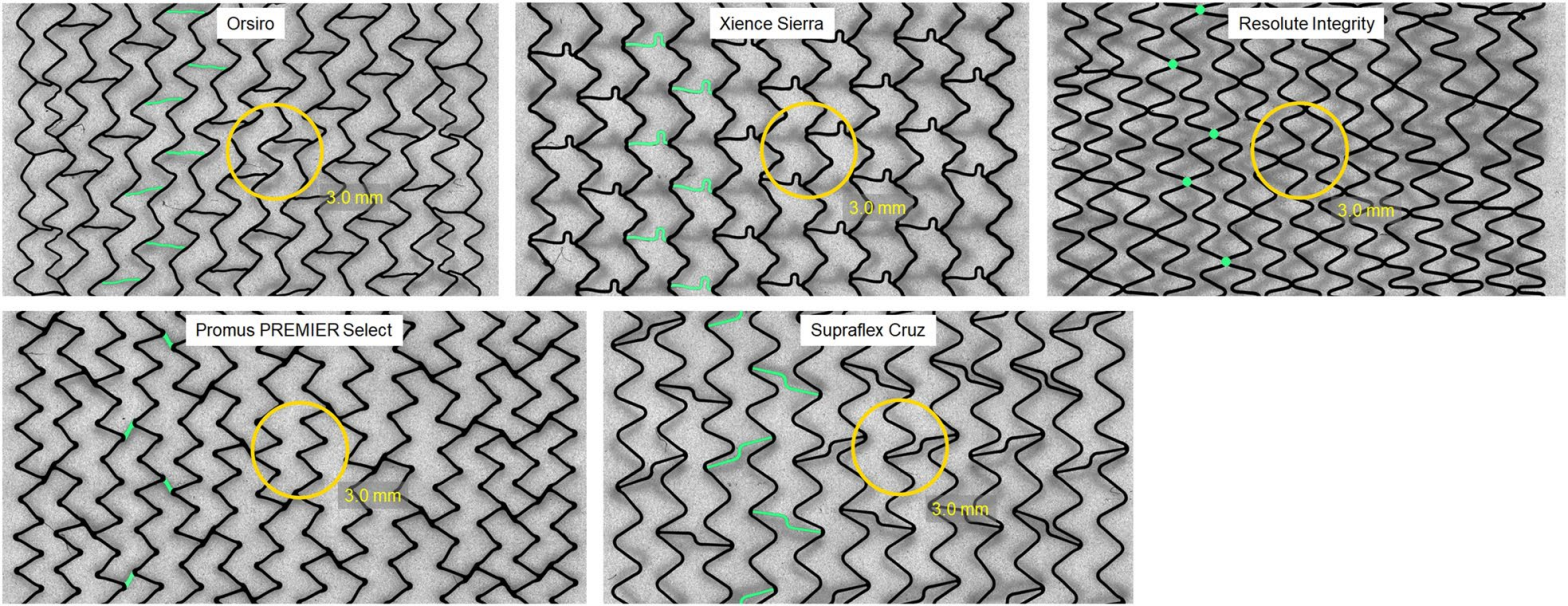
Unrolled lateral strut pattern of the analyzed contemporary stent designs obtained from high-resolution line scans. Connectors between three strut rings are highlighted in green. The yellow circle illustrates a side-branch of 3.0 mm in diameter

The overall view of the high-resolution line-scan images illustrates the arrangement of strut rings and connectors, which is distinctive for each stent design and, thus, its mechanical properties (Fig. 4: unrolled lateral strut pattern of the analyzed contemporary stent designs obtained from high-resolution line scans. Connectors between three strut rings are highlighted in green. The yellow circle illustrates a side-branch of 3.0 mm in diameter.

Of the five stent platforms investigated, four of them, namely Orsiro, Xience, Promus PREMIER Select, and SupraFlex Cruz, are arranged in a regular design pattern following dilatation at NP. The strut patterns of both Orsiro’s end and of the distal Promus PREMIER Select’s end were denser, thereby resulting in smaller stent cells. Of note, the strut rings were linked by three to four connectors in Orsiro, three connectors in Xience, two to three connectors in Resolute Integrity, and only two connectors in Promus and SupraFlex Cruz.

### Stent measurement/cell design and size

**Fig. 5:**
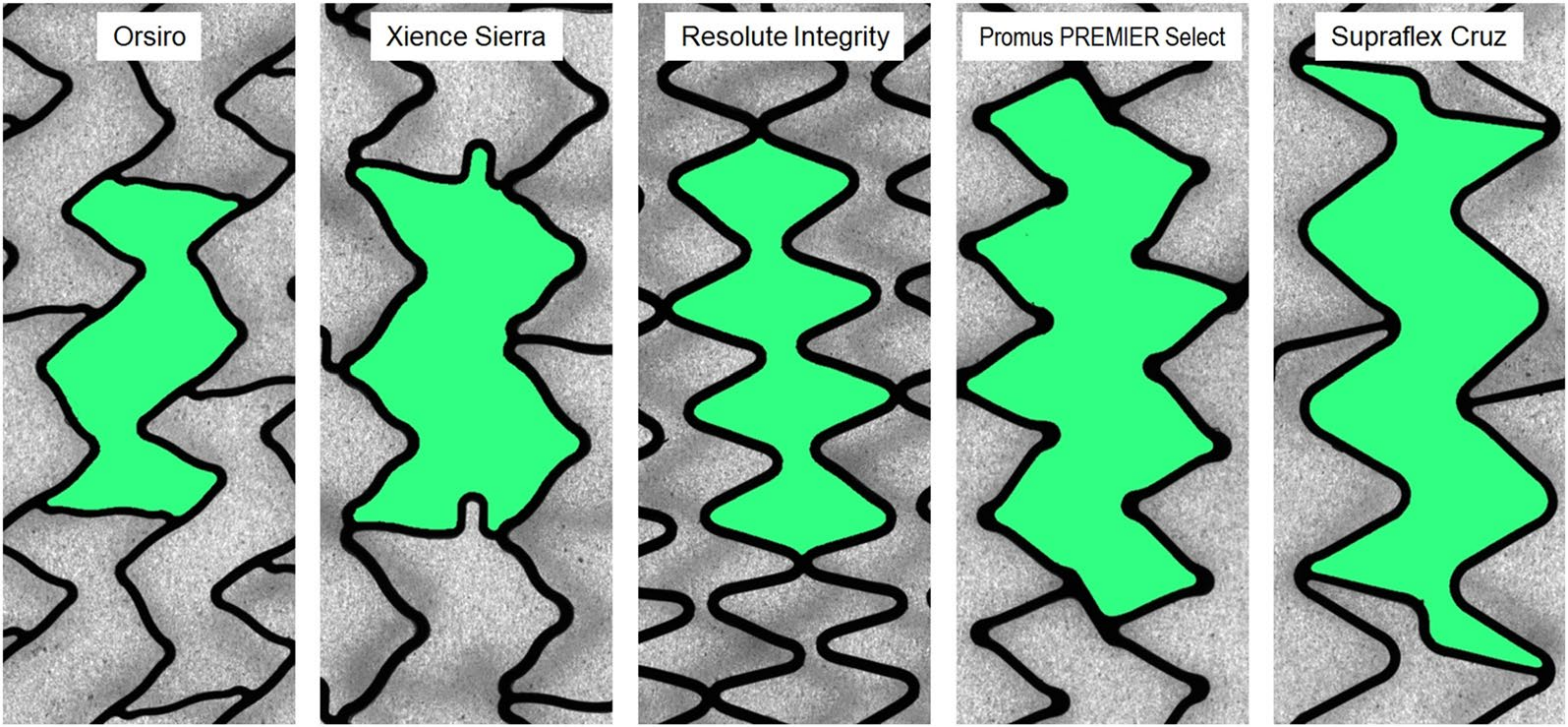
Cell design of the stent platforms expanded at nominal pressure. The same scale has been applied for the five images. Cells are represented in green

**Fig. 6:**
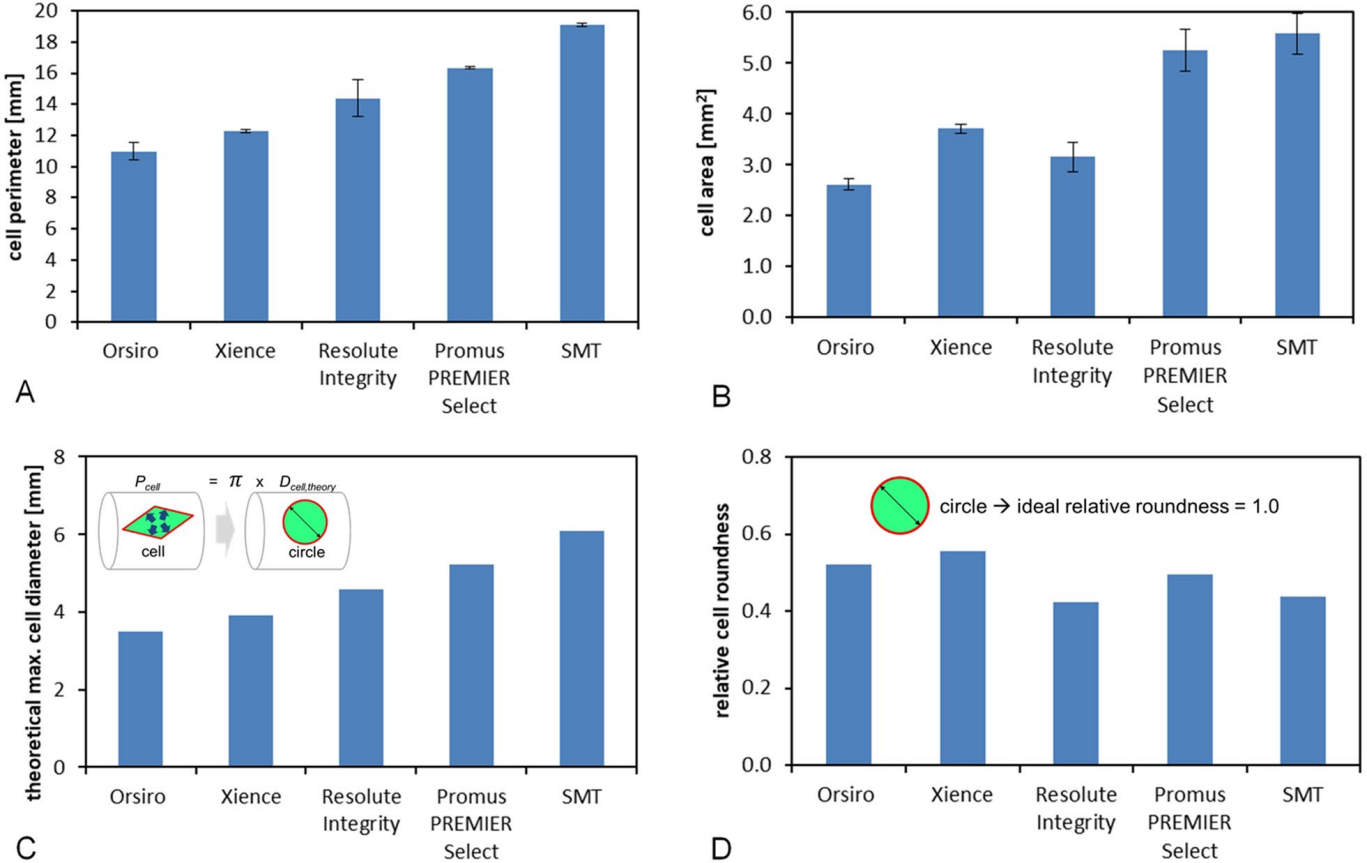
Strut pattern measurement by high-resolution line scan: **a** cell perimeter, **b** cell area, **c** derived theoretical cell diameter, and **d** relative roundness of stent cell

Both cell design and size affect the side-branch accessibility and post-dilatation capacity. A representative cell of each stent platform, obtained from high-resolution line scans, is plotted in Fig. 5. For quantitative comparison, 10 cells of each stent platform, which had previously been dilated at NP in a phantom vessel, were investigated by measuring cell perimeter *P*_cell_. In addition, the cell perimeter was employed for calculating the theoretical maximum cell diameter *D*_cell,theory_ (Fig. 6).

The stent cell perimeter was proven highly variable among the stent platforms tested. Orsiro displayed the lowest cell perimeter and SupraFlex Cruz the largest (10.97 ± 0.55 mm vs. 19.10 ± 0.11 mm). Furthermore, Resolute Integrity exhibited the highest standard deviation of cell perimeter (14.88 ± 1.31 mm), which quantifies the strut pattern’s irregularity level post-deployment.

The theoretical cell diameter limit can easily be calculated based on the stent’s cell perimeter, resulting in 3.5 mm for Orsiro and 6.1 mm for Supraflex Cruz. Notably, the theoretical limit of all stent platforms is as high as or even well above the cut-off diameter of each medium workhorse design.

To quantify the stent cell’s shape, the fluid mechanical concept of hydraulic diameter was applied:

$$D_{{\text{hyd, cell}}} = \frac{{4 \cdot A_{{{\text{cell}}}} }}{{P_{{{\text{cell}}}} }},$$

where *A*_cell_ defines the cross-sectional area of the stent cell divided by the perimeter *P*_cell_. Assuming that a circular stent cell is ideal for side-branch accessibility, the relative cell roundness can be calculated by referring to the cell’s hydraulic diameter *D*_hyd,cell_ with regard to its theoretical diameter *D*_cell,theory_ (Fig. 6 D).

### In vitro experiment—cell opening/side-branch expansion/post-dilatation

**Fig. 7:**
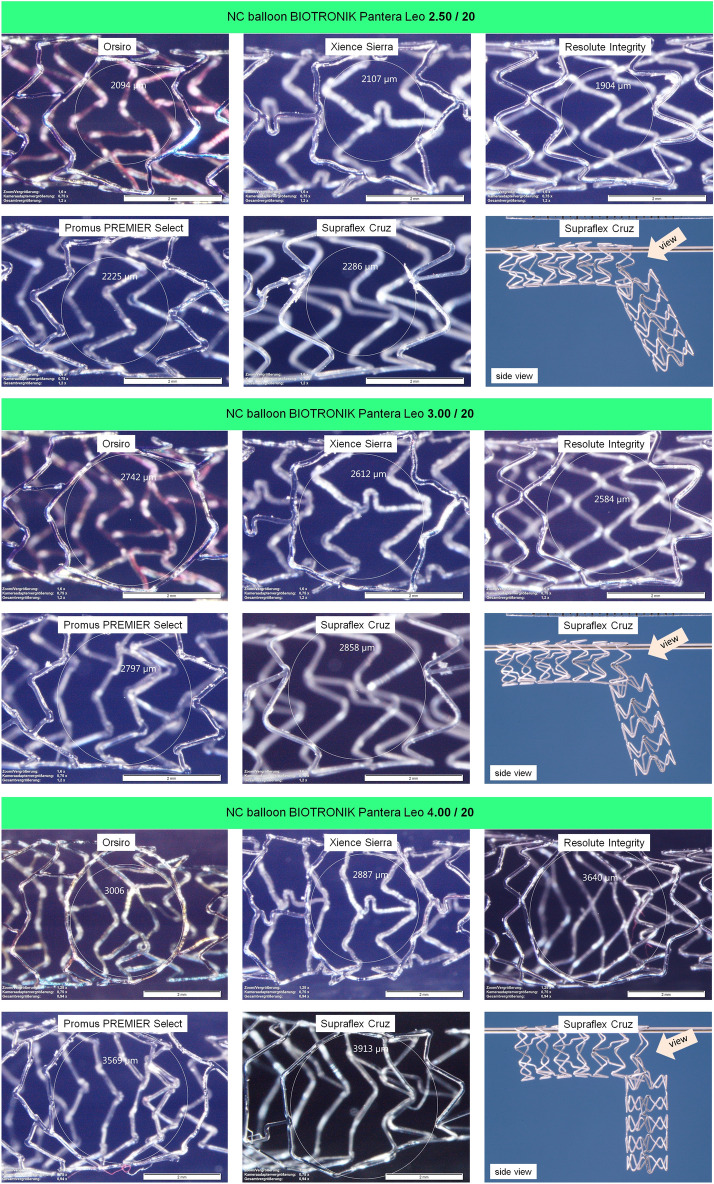
High-resolution images of stent cell after side-branch expansion using 2.50, 3.00, and 4.00 mm non-compliant balloons

**Fig. 8:**
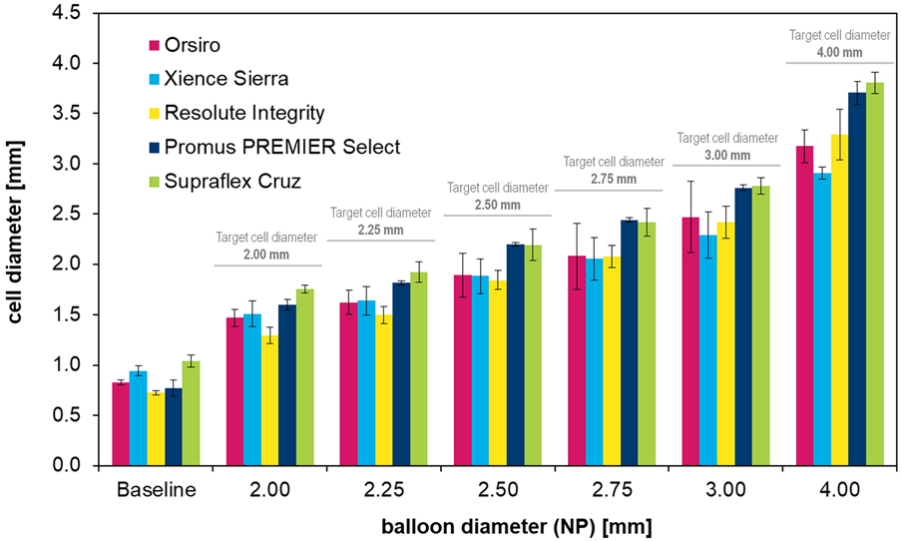
Measured cell diameter of five contemporary stent platforms following dilatation using various-sized non-compliant balloons. The marked target cell diameter is equivalent to the balloon diameter used

The side-branch expansion is performed stepwise (see Fig. 7), the measured cell diameter is depicted in Fig. 8.  Additionally, the expected cell diameter as a balloon size equivalent used is marked in the diagram.

The cell diameter enlarges with increasing balloon size, yet the expected target cell diameter is below the size of the non-compliant balloons used for each stent platform. The difference comprised between 5% (Supraflex Cruz at 4.0 mm) and 35% (Resolute Integrity at 2.0 mm). From a clinical perspective, side-branch dilatation between 2.0 mm and 3.0 mm is of particular interest. Therefore, the difference between balloon size and target side-branch diameter was calculated and averaged (2.0 mm to 3.0 mm) for each stent platform, with the following results: Orsiro: 0.59 mm ± 0.054 mm; Xience Sierra: 0.62 mm ± 0.079 mm; Resolute Integrity: 0.67 mm ± 0.055 mm; Promus PREMIER Select: 0.34 mm ± 0.069 mm; Supraflex Cruz: 0.29 mm ± 0.046 mm.

## Discussion

In this study, we have tested the side-branch expansion capacity of five commercially available stents in an in vitro model. Measurements were performed using an Olympus microscope with calibrated analysis software. As previously shown [11], we confirmed that contemporary drug-eluting stent platforms can withstand large deformations. Typically, bench tests measure the dilatation capacity of a stent segment or the overall stent structure [7, 8]. Given that side-branch expansion was performed in one single cell, our study concentrated on the interaction between balloon and stent strut within the immediate stent cell’s surroundings. Thus, the results of the stent structure overexpansion experiments, as previously published by other researchers, cannot be transposed to side-branch expansion capacity.

To investigate the influence of the stent pattern on side-branch dilatation, design features including the cell size and roundness, in addition to number of connectors, were obtained as baseline parameters for each stent deployed in the 3.0-mm phantom vessel. Cell size and cell shape were employed as criteria for side-branch accessibility. The strut pattern of Resolute Integrity exhibits the highest number of crowns. This high-dense zigzag design results in the lowest cell roundness, which, together with the small cell area for introducing guide wires, balloons, or further stents, could prove challenging when using Resolute Integrity. Despite the low cell roundness of SMT Supraflex, its huge cell area would enable good accessibility of the side-branch. The low number of connectors exerts a positive effect in this regard.

**Fig. 9:**
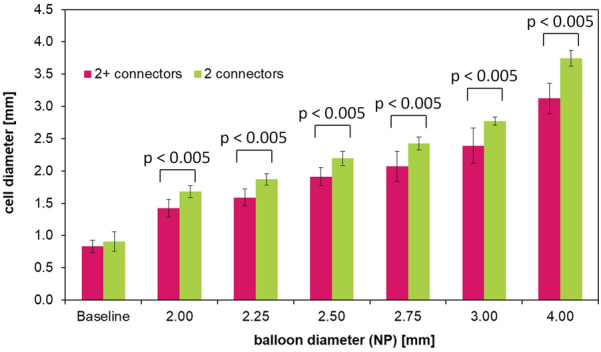
Cell diameter comparison of two-connector designs (Supraflex Cruz and Promus Premier Select) with stent platforms using more than two connectors between strut rings (2 + -connector design: Orsiro, Xience Sierra, and Resolute Integrity)

As stated by other researchers, the number of connectors strongly impacts the variety of mechanical properties of stents, including their longitudinal stiffness [12, 13] and flexibility [14]. As seen in our side-branch experiments (Fig. 8), a small number of connectors did not only result in huge cell areas, but also in larger cell diameters after side-branch expansion. When comparing two-connector designs (as used for Supraflex Cruz and Promus Premier Select) with stent platforms exhibiting more than two connectors between strut rings, a significant difference in dilatation behavior was observed (cell diameter following side-branch expansion: 2 + -connector design vs. 2-connector design: p < 0.005) (see Fig. 9).

Although the zigzag design of the strut pattern tested here can possibly be deformed without fracture, this does not necessarily mean excessive side-branch dilatation to be safe [11]. The high local strains of the stent struts may compromise the drug coating’s integrity [8, 11, 15, 16].

In our experiments, we demonstrated that stents’ side-branch expansion capacity significantly varied depending on the balloon used. To reach the target diameter within the side-branch cell area, the balloon employed had to be significantly oversized in all analyzed stents. Non-achievement of the desired diameter is thought to result from stent compliance [9] and local balloon pinching [5]. The stent individual oversize rates are provided in Table 2. Notably, the average differences between the attained cell size and target side-branch diameter were not related to the Max ID given by the manufacturer (Fig. 2).

**Table 2:** Difference between cell size attained and target side-branch diameter

Target vessel diameter = balloon diameter	Difference between attained cell size and target side-branch diameter				
	Orsiro	Xience Sierra	Resolute Integrity	Promus PREMIER Select	Supraflex Cruz
2.00	27% (0.53 mm)	24% (0.49 mm)	35% (0.70 mm)	20% (0.40 mm)	12% (0.24 mm)
2.25	28% (0.63 mm)	27% (0.61 mm)	33% (0.75 mm)	19% (0.43 mm)	15% (0.33 mm)
2.50	24% (0.61 mm)	25% (0.62 mm)	26% (0.66 mm)	12% (0.30 mm)	12% (0.31 mm)
2.75	24% (0.67 mm)	25% (0.70 mm)	25% (0.67 mm)	11% (0.31 mm)	12% (0.33 mm)
3.00	18% (0.53 mm)	24% (0.71 mm)	19% (0.58 mm)	8% (0.24 mm)	7% (0.22 mm)
Average difference	0.59 mm	0.62 mm	0.67 mm	0.34 mm	0.29 mm

To dilate the stent cell to the desired target vessel diameter of the side-branch, an overexpansion was necessary for all analyzed stent platforms. Therefore, the cardiologist had to precisely know the overexpansion values in view of proper lesion treatment. If the side-branch cell is not adequately prepared, the stent located within the side-branch is at risk of over-expanding, especially in the ostium region. This applies to all bifurcation techniques. Given this case, the side-branch stent might not achieve complete apposition to the vessel wall at the ostium. In various studies, poor stent expansion to the vessel wall was reported to be among the main causes of major adverse cardiovascular events (MACE) and in-stent thrombosis cases [17, 18]. As generally accepted, stent struts can alter blood flow in terms of wall shear stress, increased shear rate, and relative residence time [19]. Yet, altered blood flow can be detrimental while mechanically stimulating the coagulation cascade activation, which can lead to thrombus formation [20]. In bifurcation lesions, overhanging struts protruding into the ostium are, therefore, thought to act as a focal point of thrombus formation [21].

Caution should be exercised when choosing a larger balloon to match cell diameter and side-branch diameter. The observed balloon constriction is locally limited to the stent strut area. The balloon’s distal end within the side branch could reach the balloon target diameter with NP, thereby resulting in a strong vessel overexpansion.

## Conclusion

Careful selection of both stent platform and balloon may significantly impact procedural outcomes. Bench test studies have provided major information that may be instrumental for carefully selecting the appropriate size of contemporary drug-eluting stents based on their design [11].

Stent selection based on stent model design may prove critical to optimize results and ensure full stent expansion, particularly in terms of bifurcation treatments [7].

While in vitro measurements may not accurately replicate the stent mechanical behavior in vivo, they provide reasonable estimates to support operators’ decision-making in selecting instrumentation [9, 22].

A better understanding of stent designs could render bifurcation stenting both more safe and more efficient. Manufacturers are thus encouraged to provide further information on stent model design and labeled side-branch capacity on their packaging information and instructions for use, even if the device is not explicitly indicated for use in bifurcation stenting, similarly to the compliance chart and burst pressure data that are currently being provided [5].

Based on our study results, we recommend that the stent’s side-branch expansion capacity should be taken into account with respect to the bifurcation technique to be used. This study may also encourage stent manufacturers to routinely provide these measured variables for all stents. This is paramount, given that all commercially available balloon-expandable coronary stents, including those not tested in our study, were previously used to treat bifurcation lesions.

## Limitations

The measurements were performed in vitro*,* and we were thus not able to simulate the complex situation of bifurcation stenosis in humans. This study sought to describe the mechanical properties of coronary stents with regard to their side-branch expansion capacity.

Bench testing may not accurately predict stent behavior in humans. In particular, in vitro deployment without the arterial wall constraining the stent can only provide an approximation of the in vivo behavior and stent–artery response during stent deployment.

As there were only three stents tested for each of the five contemporary stent platform designs, the resulting sample size appears to be rather small. However, variations in the stents’ technical performance are considered to be small compared with variations in anatomic and pathophysiologic conditions. Therefore, we are convinced that the mechanical behavior is well described by the presented test results.

Furthermore, we only tested the 3.0-mm stent diameter of each platform. Nevertheless, this is a very commonly used stent size. In addition, the comparative results are likely to be similar for different stent diameters, provided that similar stent designs are used. Given this context, it must be noted that each stent platform is available in different sizes, which may differ in design features, including strut thickness or crown numbers. Different results can be expected when using other stent designs. Yet, the included stent designs represent the state of the art in coronary stents.

## Impact on daily practice

Percutaneous coronary interventions (PCI) of bifurcation stenoses are still challenging. To obtain side-branch access, a single stent cell needs to be expanded by balloon inflation. As there is no information available regarding the stents’ expansion behavior, we aimed to evaluate the DES designs with regard to their side-branch expansion behavior. By inflating different high-pressure NC balloons, all tested contemporary DES platforms were shown to be able to withstand large deformations, whereas the DES design did impact the expansion capacity. Furthermore, the stent cell diameter attained was below the balloon diameter at NP. As a result, we recommend that the stent cell expansion be considered in stent selection, as this information must be known for proper stent preparation in bifurcation interventions.

## Data Availability

The datasets during and/or analyzed during the current study available from the corresponding author on reasonable request.
